# Improved recovery from limb ischaemia by delivery of an affinity-isolated heparan sulphate

**DOI:** 10.1007/s10456-018-9622-9

**Published:** 2018-05-18

**Authors:** Selina Poon, Xiaohua Lu, Raymond A. A. Smith, Pei Ho, Kishore Bhakoo, Victor Nurcombe, Simon M. Cool

**Affiliations:** 1 Glycotherapeutics Group, Institute of Medical Biology, Agency for Science, Technology and Research, 8A Biomedical Grove, Immunos #06-06, Singapore, 138648 Singapore; 2 Department of Surgery, Yong Loo Lin School of Medicine, NUS and Department of Cardiac, Thoracic, Vascular Surgery, National University Health System, Singapore, Singapore; 3 Translational Molecular Imaging Group, Singapore Bioimaging Consortium, Agency for Science, Technology and Research, Singapore, Singapore; 4 Department of Biochemistry, Yong Loo Lin School of Medicine, National University of Singapore, Singapore, Singapore; 5 Department of Orthopaedic Surgery, Yong Loo Lin School of Medicine, National University of Singapore, Singapore, Singapore; 6 Lee Kong Chian School of Medicine, Nanyang Technology University – Imperial College London, Singapore, Singapore

**Keywords:** Angiogenesis, VEGF, Blood vessel formation, Glycosaminoglycan, Vascular insufficiency

## Abstract

Peripheral arterial disease is a major cause of limb loss and its prevalence is increasing worldwide. As most standard-of-care therapies yield only unsatisfactory outcomes, more options are needed. Recent cell- and molecular-based therapies that have aimed to modulate vascular endothelial growth factor-165 (VEGF_165_) levels have not yet been approved for clinical use due to their uncertain side effects. We have previously reported a heparan sulphate (termed HS7) tuned to avidly bind VEGF_165_. Here, we investigated the ability of HS7 to promote vascular recovery in a murine hindlimb vascular ischaemia model. HS7 stabilised VEGF_165_ against thermal and enzyme degradation in vitro, and isolated VEGF_165_ from serum via affinity-chromatography. C57BL6 mice subjected to unilateral hindlimb ischaemia injury received daily intramuscular injections of respective treatments (*n* = 8) and were assessed over 3 weeks by laser Doppler perfusion, magnetic resonance angiography, histology and the regain of function. Mice receiving HS7 showed improved blood reperfusion in the footpad by day 7. In addition, they recovered hindlimb blood volume two- to fourfold faster compared to the saline group; the greatest rate of recovery was observed in the first week. Notably, 17% of HS7-treated animals recovered full hindlimb function by day 7, a number that grew to 58% and 100% by days 14 and 21, respectively. This was in contrast to only 38% in the control animals. These results highlight the potential of purified glycosaminoglycan fractions for clinical use following vascular insult, and confirm the importance of harnessing the activity of endogenous pro-healing factors generated at injury sites.

**Electronic supplementary material:**

The online version of this article (10.1007/s10456-018-9622-9) contains supplementary material, which is available to authorized users.

## Introduction

Vascular insufficiency affects one in ten people over 70 years of age and one in six over 80 [1]. With age as a major contributing factor, the number of people diagnosed with peripheral arterial disease (PAD) increased by over 23% in the decade between 2000 and 2010 [1]. Current treatment options include either changes in lifestyle for patients experiencing intermittent claudication or surgical intervention such as open surgical bypass or catheter-based revascularization for patients at risk of limb loss. With concerns growing around the long-term patency of surgical procedures [2], and the ongoing failure of procedural revascularization in a significant proportion of patients [3], clinicians are also focusing efforts on alternative strategies. Novel attempts to induce new vessel formation at sites of tissue ischaemia have concentrated on the use of O_2_ therapy [4], stem cell [5] or growth factor therapy [6]. They all aim to increase the local concentration of growth factors to stimulate angiogenesis. More recently, restoring balance in the glycosaminoglycan (GAG) fraction of vessel extracellular matrix (ECM) has proven effective for the induction of new vessels in embryonic models [7]. Such GAG-based treatments are novel because they act by sequestering, and then bio-activating powerful endogenous mitogens naturally produced at sites of tissue ischaemia to promote the formation of new vessels, all without the risks inherent with exogenous growth factor therapy.

Angiogenesis is a complex process that involves temporal regulation of growth factor signalling to initiate neovascularization and remodelling at the compromised site. The most actively studied growth factor for angiogenic therapy has been vascular endothelial growth factor-A (VEGF-A) [8]. Neovascularization in the ischaemic limb can be attenuated with the use of neutralising antibodies against VEGF [9], highlighting its critical role during vessel recovery in the early stages of revascularization. Most investigations have focused on the 165-isoform of VEGF-A (VEGF_165_) due to its intrinsic potency. While the use of VEGF-A has proven successful for treating ischaemia in animal models [10], only modest benefits have been reported in human clinical trials [11], mainly owing to the short half-life of the growth factor [12, 13] when confronted with fluctuations in inflammatory state, pH, temperature, oxidation states and proteolytic factors within sites of damage [14]. In attempts to overcome the poor pharmacokinetics, multiple large doses of growth factors are frequently administered, which often result in unwanted toxicity and adverse off-target events [15].

The binding of VEGF to receptors on endothelial cells to initiate the angiogenesis cascade [16] is dependent on particular forms of its carbohydrate cofactor, heparan sulphate (HS) [17]. Such a crucial role suggests the possibility of developing these sugars for angiogenic therapy. However, targeting the binding of HS to particular proteins is necessary for driving desirable outcomes, because the inherent heterogeneity of pericellular HS leads to the binding of a plethora of factors that can otherwise disrupt angiogenic progression. We previously reported a VEGF_165_-binding HS (HS7) that was isolated via affinity-chromatography using the heparin-binding domain of VEGF_165_ as an affinity substrate [18]. HS7 demonstrated increased affinity for VEGF_165_ and potentiated endothelial cell proliferation and vessel tube formation in an embryonic model. The most striking observation was the ability of HS7 alone to promote blood vessel formation in the chick embryo chorioallantoic membrane assay without the addition of exogenous VEGF_165_ [18]. Here, we sought to determine whether VEGF_165_ could be protected from thermal and enzymatic degradation by HS7. In a hypoxic environment, where VEGF expression is elevated to promote angiogenesis and reperfusion of blood supply to the injured site, one of the factors determining the extent of the angiogenic response is its stability. VEGF is very susceptible to both thermal and enzymatic degradation in vivo. Brandner et al. have previously demonstrated that VEGF can be protected against thermal degradation by heparin [19].

To further exemplify the proangiogenic potential of HS7, we induced hindlimb ischaemia in mice and investigated the ability of HS7 to restore blood flow. Our results demonstrate that HS7 by itself can restore blood volume at ischaemic sites by increasing blood vessel density. The results support the continued development of glycosaminoglycans as a means of therapeutic angiogenesis.

## Methods

### Materials

HS7 used in this paper was isolated from crude porcine mucosal HS (HS^pm^, Lot# HO-10697; Celsus Laboratories, Cincinnati, OH) using the methods previously described [18]. Recombinant human VEGF_165_ (VEGF_165_) and Toll-like receptor-4 (TLR4), and biotinylated antibody against VEGF_165_ were from R&D Systems (Minneapolis, MN). Horseradish peroxidase (HRP)-conjugated streptavidin was from Invitrogen. Plasmin, general chemicals and reagents were purchased from Sigma-Aldrich. Human umbilical vein endothelial cells (HUVECs) (Merck Millipore) were provided at passage 1 and maintained with the EndoGRO™-Low Serum culture media kit (Merck Millipore). HUVECs from passages 2–8 were used for all experiments.

### Thermal degradation and plasmin proteolysis

To investigate thermal stability, 5.2 mmol/L VEGF_165_ was pre-incubated with or without respective HS (1 µg/µl) on ice for 10 min. The reactions were then transferred to 37 °C for further incubation, or − 80 °C for subsequent freeze–thaw treatment. For reactions incubated at 37 °C, aliquots of each reaction were collected at stipulated time points and stored at − 80 °C.

In addition to thermal degradation, VEGF_165_ is also susceptible to enzymatic digest by plasmin in vivo. To determine the stability of VEGF_165_ against plasmin digest, 5.2 mmol/L VEGF_165_ was pre-incubated on ice for 10 min with or without respective HS (1 µg/µL). Plasmin was added to the reactions (final activity 0.5 mU/µL) and then further incubated for 4 h at 37 °C.

At the end of the respective assays, 4× native gel loading buffer (40 mmol/L Tris–HCl pH 8, 400 mmol/L KCl, 40% *v*/*v* glycerol, 0.4% *v*/*v* NP-40) was added to the thermal and proteolytic degradation reactions, resolved on 4–12% Bis-Tris gelsNuPAGE, Novex, Life Technologies) and blotted onto nitrocellulose membranes. Membranes were blocked and incubated with a biotinylated anti-VEGF antibody, followed by HRP-conjugated streptavidin. Immunoreactive bands were visualised using the LumiGLO® Chemiluminescent Substrate Kit.

### VEGF-VEGFR2 signal transduction

HUVECs were seeded in 12-well plates at a density of 190,000 cells per well and cultured in EndoGRO™-Low Serum complete culture media kit lacking HS, rEGF and LS-growth supplement for 24 h. VEGF_165_ (treated as described in *Thermal degradation and plasmin proteolysis*) was added to cells and incubated for 10 min at 37 °C. Cells were washed with ice-cold 1× PBS and lysed in ice-cold RIPA lysis buffer containing protease inhibitor cocktail and 2 mmol/L sodium orthovanadate. The lysate was collected and incubated on ice for 20 min and clarified. Total protein content was determined using the BCA quantification method with the Pierce™ BCA Protein Assay Kit (Thermo Scientific). For each reaction, 10 µg of protein was resolved on 4–12% Bis-Tris gels under reducing conditions and blotted onto nitrocellulose membranes. Membranes were subsequently blocked and probed with specific primary antibodies (Cell Signaling Technology Inc.) followed by appropriate HRP-conjugated secondary antibodies (Jackson Immunoresearch). Immunoreactive bands were visualised using the LumiGLO® Chemiluminescent Substrate Kit (KPL, USA).

### Cell proliferation

The BrdU Cell Proliferation Kit (Roche) was used to assay cell proliferation. HUVECs were seeded in 96-well plates at a density of 50 cells/mm^2^ (square millimetre) and cultured in EndoGRO™-Low Serum complete culture media kit lacking HS, rhEGF and LS-growth supplement for 24 h. VEGF_165_ (treated as described in *Thermal degradation and plasmin proteolysis*) was added to cells and incubated at 37 °C. After 24 h, BrdU was added to cells and incorporated for 24 h. BrdU detection was performed according to the manufacturer’s protocol.

### Affinity fractionation using HS-tagged chromatography columns

The interaction of HS with growth factors in serum was investigated using a modified affinity fractionation protocol. To prepare the column, lyophilised cyanogen bromide-activated Sepharose 4B (CNBr-Sepharose) (GE Healthcare) was rehydrated in 1 mmol/L HCl. The CNBr-Sepharose slurry was incubated with HS in binding buffer (100 mmol/L NaHCO_3_, 500 mmol/L NaCl, pH 8.3) at a ratio of 2:1 HS to CNBr-Sepharose to obtain HS-tagged Sepharose beads that were loaded onto chromatography columns and washed with wash buffer (1× PBS, 1 mol/L NaCl), followed by equilibration buffer (50 mmol/L Tris, 200 mmol/L NaCl, 2 mmol/L CaCl_2_, 2 mmol/L MgCl_2_, pH 7.4). The fractionation sample was prepared by adding 500 ng VEGF_165_ and 500 ng BMP-2 (bone morphogenetic protein-2) to fetal bovine serum (FBS), then diluted in equilibration buffer. The samples were added to the columns (HS7-tagged or HS^ft^-tagged columns) and washed with equilibration buffer via gravity-flow. A first elution was done using Elution Buffer 1 (50 mmol/L Tris, 500 mmol/L NaCl, 2 mmol/L CaCl_2_, 2 mmol/L MgCl_2_, pH 7.4) to remove weak binding proteins to the HS-tagged column. A second elution was then performed using Elution Buffer 2 (50 mmol/L Tris, 1 mol/L NaCl, 2 mmol/L CaCl_2_, 2 mmol/L MgCl_2_, pH 7.4) to elute proteins that firmly bound to the column. Samples eluted with Elution Buffer 2 were prepared for immunoblotting as previously described using native gel loading buffer.

### Surface-plasmon resonance (SPR)-based measurement of protein binding to heparin-coated SA sensor chip

Protein binding to a streptavidin (SA) sensor chip was performed as previously described [18]. Briefly, biotinylated heparin was immobilised onto a streptavidin (SA) sensor chip. Protein binding to the heparin support was detected using a BIACORE T100 surface-plasmon resonance instrument as per the manufacturer’s protocols (GE Healthcare, Sweden). Toll-like receptor 4 (TLR4) was prepared in HBS-EP running buffer (10 mM HEPES, 150 mM NaCl, 0.1% *v*/*v* Tween-20) and applied to the sensor chip at a flow rate of 30 µL/min for 120 s, followed by washing with running buffer for 600 s. The sensor chip was regenerated between consecutive applications of TLR4 at different concentrations. VEGF_165_ was applied separately as a comparison.

### Signal transduction in RAW264.7

RAW264.7 cells were cultured in Dulbecco’s Modified Eagle Medium (DMEM, high glucose, pyruvate preparation, Gibco™, Thermo Fisher Scientific) supplemented with 10% *v*/*v* FBS and 100 µg/mL streptomycin and 100 IU/mL penicillin. For signal transduction, RAW264.7 were seeded in 6-well plates at a density of $2 \times {10^6}$ cells per well. Lipopolysaccharide (LPS) at 50 ng/mL, or HS at 10 µg/mL were added to cells and incubated for 20 min at 37 °C. Cells were then washed and lysate was collected and quantified as described in the section on “*VEGF-VEGFR2 signal transduction*”. For each treatment condition, 20 µg of lysate was resolved on 4–12% Bis-Tris gels under reducing conditions, blotted onto nitrocellulose membranes, and probed with specific antibodies followed by appropriate HRP-conjugated secondary antibodies. Immunoreactive bands were visualised using the LumiGLO® Chemiluminescent Substrate Kit.

### Efficacy study of HS7 in murine model of hindlimb ischaemia

All animal-related procedures were in accordance with Institutional Animal Care and Use Committee guidelines at the Biological Resource Centre, A*STAR, Singapore (IACUC #130842). Unilateral hindlimb ischaemia was induced in the right hindlimb of C57BL/6N mice (male, 10 weeks old, each weighing 20–25 g) as described previously [20]. Male mice were chosen to exclude the effect of hormonal influences in the angiogenic healing response. Also, the C57BL/6 strain was shown to exhibit elevated expression of the murine VEGF-A-164 isoform following ischaemia, which shares 89% homology with corresponding regions of human VEGF_165_ and could stimulate HUVEC proliferation [8, 9]. Briefly, the mice were anaesthetised with an intraperitoneal injection of Ketamine (150 mg/kg) and Xylazine (10 mg/kg). A 1-cm incision was made in the right hindlimb from the medial thigh to the knee. The external iliac artery was isolated, ligated twice using 7/0 polypropylene suture (Premilene, Braun, Melsungen AG) and then transected between the two ligations. The mice received subcutaneous injections of Enrofloxacin (10 mg/kg) once daily for 5 days and of Buprenorphine (0.1 mg/kg) twice daily for 3 days after the surgery. The animals were divided into four treatment groups. One day after surgery and for a further 7 days, animals were injected daily with 3 µg HS7 (*n* = 8), 30 µg HS7 (*n* = 8) or 30 µg flowthrough HS (HS^ft^, *n* = 8) intramuscularly at three different sites on the operated limb (vastus lateralis, vastus medialis and gastrocnemius). All HSs were delivered in 60 µL of PBS. Controls (*n* = 8) received PBS (60 µL) only. Postoperative loss and gain of function, during the treatment and recovery phases were recorded and categorised numerically from 1 to 4, with 1 showing loss of function and 4 showing recovery of function (Table 1). A list of animals is shown in the Supplemental Table (Table S1).

**Table 1:** Hindlimb functional score

4	Full recovery
3	Able to flex toes, but lacks fine motor control
2	Able to support weight on the hindlimb
1	Dragging hindlimb

### Laser Doppler flow imaging (LDI)

Blood perfusion in the plantar foot was assessed using a PeriScan PIM 3 laser Doppler system (Perimed AB, Sweden) preoperatively, postoperatively and on days 3, 7, 14 and 21. Animals were anaesthetised with 2% isoflurane and placed in a prone position connected to a continuous flow of isoflurane (1–3%) for the duration of the scan. Images were analysed using PIMSoft software (Perimed AB, Sweden). The footpad was used as the region of interest; the perfusion rate in the footpad of the right (ligated) limb was normalised to the contralateral (non-ligated) limb.

### Magnetic resonance angiography (MRA)

The hindlimb vasculature of the animals was acquired using time-of-flight (TOF) magnetic resonance angiography (MRA) with a Bruker Biospec 9.4T scanner (Bruker, Germany). A flow compensated gradient-echo TOF protocol, with a spatial resolution of 0.109 millimetres per pixel (mm/pixel) × 0.109 mm/pixel, 0.35 mm slice thickness and 150 slices, was applied at ultra-high field to characterise the vascular signal above the signals originating from the surrounding stationary tissues. Signals originating from the stationary tissues were saturated with very short repetition time such that the longitudinal magnetization of these tissues did not have time to recover, thereby weakening their signal. This favours the inflow effect.

The MRA data were analysed using ImageJ software. Blood vessels were segmented by thresholding. The total volume of blood was calculated by multiplying the total vessel area by the slice thickness. 80 slices covering the start of the thigh to the lower calf were used for blood volume quantification. Image slices were stacked using Onis 2.5 (DigitalCore Co. Ltd., Japan) to create three-dimensional views of the vasculature.

### Immunohistochemistry and histomorphometric analysis

Quadriceps, hamstring and gastrocnemius tissues were harvested on day 8 post-surgery. Tissues were fixed in 10% neutral buffered formalin and embedded in paraffin. Serial transverse sections from all tissues were obtained and stained for visualisation of von Willebrand factor (vWF) with rabbit anti-human vWF antibody (Dako, USA) using the LEICA Bond Auto Stain according to the manufacturer’s instructions, and also for haemotoxylin and eosin (H&E) staining for visualisation of muscle morphology. Transverse sections from quadriceps tissue were obtained and stained for visualisation of α-smooth muscle actin (αSMA) using rabbit anti-αSMA antibody (Abcam, Catalogue ab5694) following standard immunohistochemistry protocols. Images for quantification of vWF-stained and αSMA-stained vessels were captured under brightfield using the Metafer 4 automated captured system (MetaSystems, Germany). Vessels positive for vWF and αSMA were counted from six random fields of view per section and represented as the number of vWF-positive (vWF^+^) or αSMA^+^ cells ± standard deviation per square millimetre of area quantified. For display purposes only, representative images were captured on the ZEISS AxioImage Z1 (ZEISS, Germany) and uniform adjustments were made on ImageJ to enhance contrast. The number of regenerating myofibres, defined as having a centrally located nuclei [21], was counted from H&E sections. Data were presented as the mean number of regenerating myofibres ± standard deviation per square millimetre of area quantified. All measurements were made on unprocessed images.

### Statistical analysis

Data on proliferation were reported as the mean ± standard deviation of three independent experiments. Data on LDI and MRA were reported as the mean fold-change ± standard deviation. Statistical analyses were performed with GraphPad Prism 7.0a (GraphPad Software Inc., San Diego, CA). Unpaired *t* test, or one-way and two-way ANOVA with Tukey’s multiple comparisons were performed where appropriate.

## Results

### Growth factor stability

**Fig. 1:**
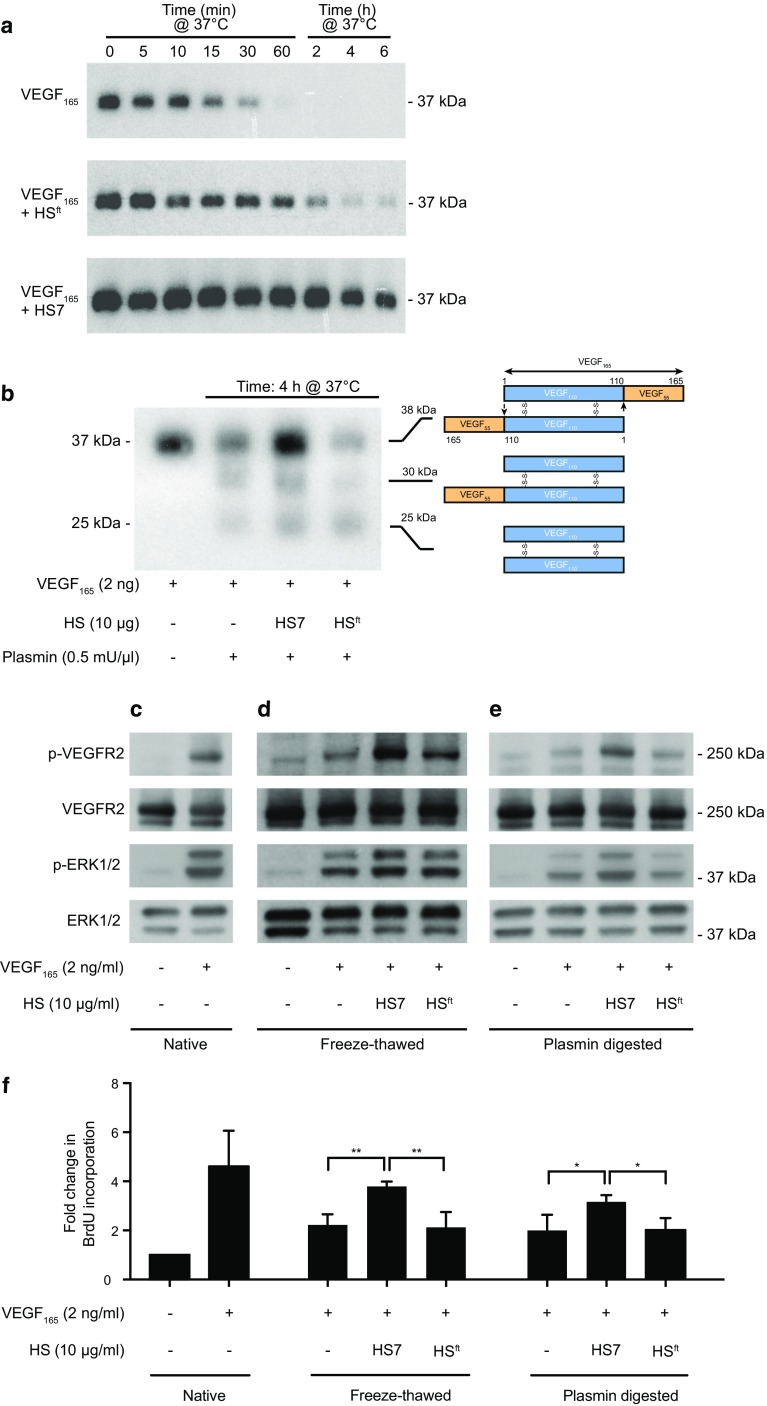
In vitro analyses of HS7 activity **a** Thermal degradation of VEGF_165_ complexed with HS variants. Representative immunoblots showing the degradation of VEGF_165_ complexed with HS variants over time at 37 °C. **b** Representative immunoblot showing plasmin proteolysis of VEGF_165_ complexed with HS variants for 4 h at 37 °C. The schematic diagram to the right of the immunoblot shows the VEGF_165_ homodimer, VEGF_165_/VEGF_110_ heterodimer and VEGF_110_ homodimer, and the approximate molecular weights they resolve to via polyacrylamide gel electrophoresis: 38, 30 and 25 kDa, respectively. The plasmin cleavage sites are indicated by arrows. Signal transduction in HUVECs induced by **c** native VEGF_165_, **d** freeze–thawed HS/VEGF_165_ complexes, or **e** plasmin-digested HS/VEGF_165_ complexes. **f** HUVEC proliferation stimulated by native, freeze–thawed or plasmin-digested HS/VEGF_165_ complexes. Immunoblots shown are representative of three independent experiments. Data for BrdU incorporation are represented as mean ± SD of fold-change calculated from three independent experiments. One-way ANOVA with Tukey’s multiple comparisons test was performed (*p < 0.05; **p < 0.01)

To assess the ability of HS fractions to maintain the stability of VEGF_165_ at physiological temperature, we incubated VEGF_165_ with or without HS variants at 37 °C and then tested for the presence of the native, homodimeric VEGF_165_ by immunoblotting. In the absence of HS, the amount of homodimeric, ~ 38 kDa VEGF_165,_ declined rapidly over time and was no longer detectable within 30 min (Fig. 1a). In contrast, VEGF_165_ incubated with HS7 at 37 °C remained detectable at all time points examined (up to 6 h). In comparison, depleted HS^ft^ was only able to maximally protect VEGF_165_ for the first 60 min.

Growth factors are susceptible to enzyme proteolysis in vivo, with VEGF_165_ particularly vulnerable to digestion by plasmin, an enzyme present in blood that is responsible for clot dissolution and extracellular matrix protein degradation [22]. The plasmin cleavage of VEGF_165_ removes its heparin-binding domain, the basic region that gives the growth factor the ability to interact with endogenous HS [23]. We, therefore, investigated the ability of HS variants to protect VEGF_165_ from proteolysis by plasmin.

Under non-reducing conditions, intact VEGF_165_ migrated to its predicted 38 kDa (Fig. 1b, first lane). Plasmin cleavage of VEGF_165_ gave rise to two additional bands at approximately 30 and 25 kDa (Fig. 1b, second lane). These corresponded to the VEGF_165_/VEGF_110_ heterodimer and VEGF_110_ homodimer, respectively (Fig. 1b). In the absence of HS, there was a decreased amount of VEGF_165_ homodimer detected after plasmin digest; in contrast, the VEGF_165_ homodimer was strongly detected after plasmin digest in the presence of HS7. Furthermore, there was considerably more VEGF_165_ homodimer present when incubated with HS7 than with HS^ft^.

### Signal transduction and proliferation

Protein denaturation and enzymatic proteolysis lead to a loss of biological activity. To investigate whether HS7 was able to maintain the biological activity of VEGF_165_ after a denaturing or proteolytic event, VEGF_165_ pre-complexed with HS variants was subjected to a freeze–thaw cycle or plasmin proteolysis, and then used to challenge HUVECs. Under native conditions (the absence of freeze–thaw or plasmin proteolysis), signal transduction induced by VEGF_165_ led to increased VEGF receptor-2 and ERK1/2 phosphorylation (Fig. 1c). Complexing VEGF_165_ with HS7 before freeze–thaw or plasmin proteolysis maintained VEGF_165_ signal transduction activity when compared to VEGF_165_ alone (Fig. 1d, e, respectively). The level of phosphorylation induced by VEGF_165_ was also increased in the presence of HS7 compared to HS^ft^ (Fig. 1d, e, respectively), an observation consistent with a previous report [18].

The ability of HS7 to stabilise VEGF_165_ against thermal or proteolytic denaturation was further investigated with a proliferation assay. Similar for signal transduction, VEGF_165_, freeze–thawed or plasmin-digested in the presence or absence of HS variants, was added to cells and proliferation measured by BrdU incorporation. Both thermal and plasmin exposure reduced the bioactivity of VEGF_165_ compared to native VEGF_165_ (Fig. 1f). In the presence of HS7, VEGF_165_ exerted greater proliferative effects on HUVECs, which was in contrast to the HS7-depleted HS^ft^ (Fig. 1f, *p* < 0.01 and *p* < 0.05, respectively).

### Binding of HS7 to VEGF_165_ in serum

**Fig. 2:**
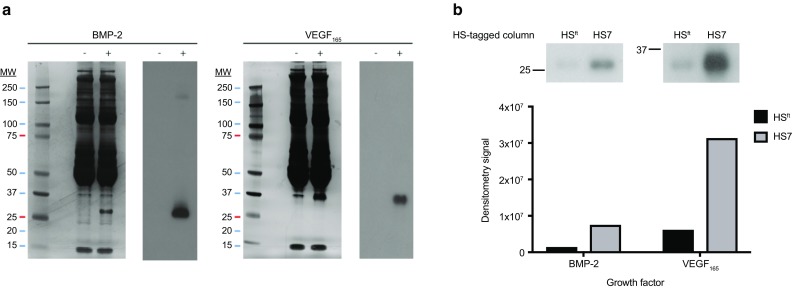
Elution of VEGF_165_ from HS-tagged columns **a** Silver-stained gel image and corresponding immunoblot of BMP-2 or VEGF_165_ containing FBS from a single experiment. The addition of BMP-2 or VEGF_165_ to FBS (denoted by ‘+’) showed a band at approximately 25 and 37 kDa on the silver-stained gel, respectively, which corresponded to the respective immunoblots at similar molecular weights. **b** Representative immunoblot of BMP-2 and VEGF_165_ eluted from HS^ft^-tagged or HS7-tagged affinity columns and its corresponding densitometry analysis (performed on ImageStudio Lite Version 5.2.5)

The in vivo environment is rich with plasma serum proteins that could potentially disrupt the interaction of HS7 with its targeted growth factor. Therefore, HS7 or HS^ft^ was used as an HS-tagged affinity-column to determine its affinity for VEGF_165_ in serum. FBS was used to simulate the in vivo condition. As the amount of BMP-2 and VEGF_165_ was low in FBS, we added exogenous BMP-2 and VEGF_165_ prior to the start of the affinity capture. Figure 2a depicts silver-stained gel images of FBS with BMP-2 and VEGF_165_ added, and the corresponding immunoblots (Fig. 2a).

Figure 2b shows representative images of BMP-2 and VEGF_165_ eluted under high salt conditions of 1 mol/L NaCl, and its corresponding densitometry analysis (ImageStudio Lite Version 5.2.5). The amount of VEGF_165_ eluted from the HS^ft^-affinity capture column was low, compared to VEGF_165_ eluted from the HS7-tagged affinity column. Densitometry revealed that HS^ft^ has low affinity for VEGF_165_ in FBS, while it bound to the HS7-tagged column with higher affinity (Fig. 2b). A replicate experiment demonstrated similar findings (Fig. S1).

### Recovery from limb ischaemia

**Fig. 3:**
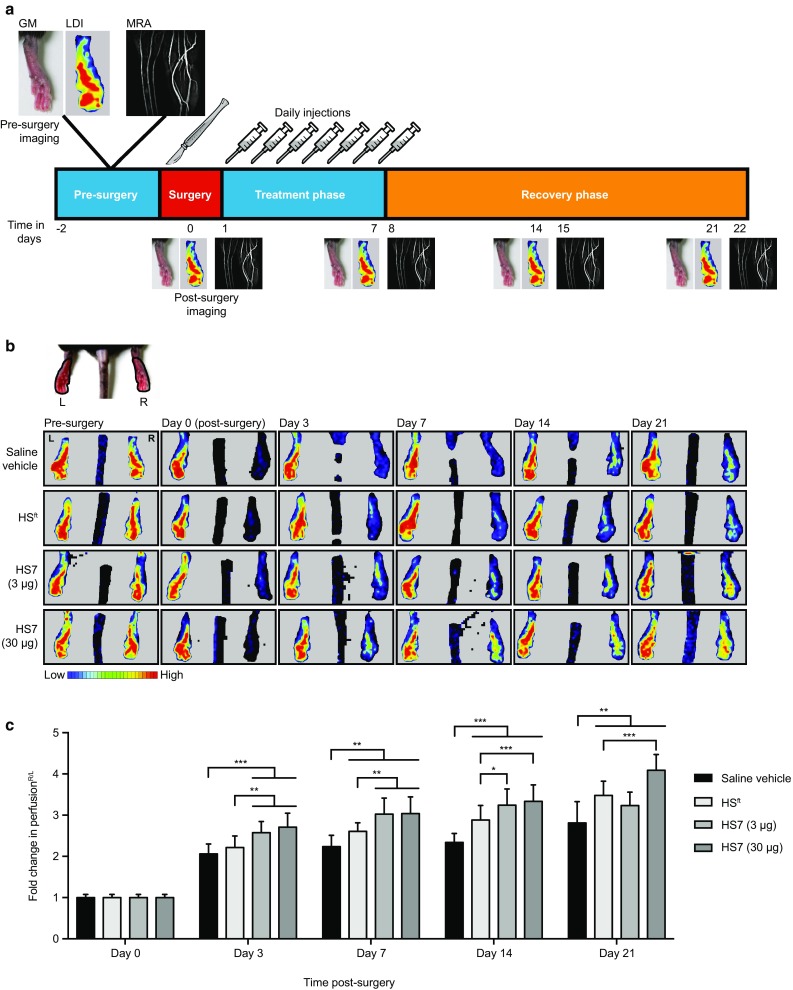
Time-line of study and LDI scanning **a** Time-line of in vivo study. *GM* gross morphology, *LDI* laser Doppler imaging, *MRA* magnetic resonance angiography. **b** The lower limbs of a mouse in a prone position were shown, with the left (L) and right (R) plantar feet indicated. Laser Doppler images shown follow this orientation. Pre-surgery, blood perfusion in both the left and right feet were similar, in contrast to post-surgery when blood perfusion to the right foot was obstructed. The time series showed the recovery of blood perfusion to the right foot. **c** Fold-change in blood perfusion normalised to day 0 (post-surgery). Two-way ANOVA with Tukey’s multiple comparisons test was performed (*p < 0.05; **p < 0.01; ***p < 0.001)

**Fig. 4:**
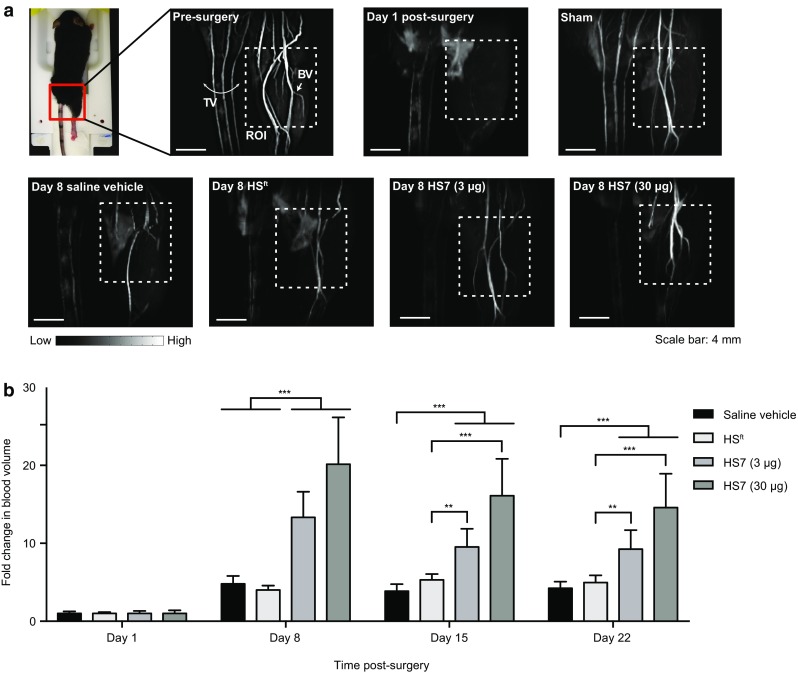
Magnetic resonance angiography **a** An image of a mouse in a prone position on a cradle was shown. The red box delineates the region of the hindlimb that was imaged. Reconstructed MRA images obtained before and post-surgery confirmed the loss of blood flow following external iliac artery ligation. Reconstructed MRA images of sham-operated mice showed no difference from pre-surgery. Representative reconstructed MRA images of mice are shown after a week of treatment (day 8). High blood flow is depicted by bright signals and low blood flow is depicted by dark signals. *TV* tail vein, *BV* blood vessel, *ROI* region of interest of blood volume quantification. **b** Fold-change in blood volume in the ischaemic hindlimb. Blood flow was measured by MRA and normalised to day 1 post-surgery levels. Two-way ANOVA with Tukey’s multiple comparisons test was performed (**p < 0.01; ***p < 0.001)

The efficacy of HS7 as a therapeutic for blood vessel repair was tested in a clinically relevant, peripheral vascular disease model. As summarised in the timeline (Fig. 3a), we ligated the external iliac artery in the right limb of C57BL/6N mice and confirmed successful ligation by LDI (Fig. 3b, Day 0 Post-surgery) and MRA (Fig. 4a, Day 1 Post-surgery), before the start of treatment. Saline, HS7 (3 or 30 µg) or HS^ft^ (30 µg) were delivered via daily intramuscular injection for one week post-surgery. Recovery was monitored over 3 weeks by LDI and MRA (Fig. 3a). Representative LDI taken of the plantar foot is presented in Fig. 3b as a time series. Representative reconstructed images of blood volume taken by MRA on day 8 are depicted in Fig. 4a.

After receiving intramuscular injections daily for 1 week post-surgery, revascularization was observed in all groups by both LDI (Fig. 3b) and MRA (Fig. 4a). However, the extent and rate of recovery observed varied across the treatment groups. Blood perfusion to the plantar foot measured by LDI showed the greatest change up to 3 days post-surgery. Although the recovery in blood perfusion at subsequent time points was gradual, treatment with 30 µg of HS7 consistently gave the highest change in blood perfusion compared to saline vehicle or HS^ft^ (Fig. 3c; **p < 0.01, ***p < 0.001). This indicated that while the rate of recovery was continuous in all animals, reperfusion was enhanced in HS7-treated animals.

Blood volume in the ischaemic limb also showed a similar recovery response as seen with blood perfusion. Day 8 angiograms revealed the presence of bright signals in the ligated limb indicating blood flow (Fig. 4a), compared to their absence from angiograms taken at day 1 post-surgery. Animals treated with HS7 had greater signals associated with the ligated hindlimb, which translated into higher volumes of blood present (Fig. 4a). This was most evident when blood volume was compared to post-surgery blood volume on day 1 (Fig. 4b). Regardless of treatment received, the greatest change in blood volume measured in the ligated limb was between days 1 and 8, after which no further increase was observed at any subsequent time point across all groups (Fig. 4b). A comparison of the recovery rate across treatment groups, however, showed that animals treated with HS7 had the greatest change in blood volume (***p < 0.001). Treatment with HS7 led to a 13- to 20-fold-change in blood volume from day 1 to 8 post-surgery, which was two- to fourfold higher than treatment with saline vehicle or HS^ft^ (Fig. 4b; ***p < 0.001).

**Fig. 5:**
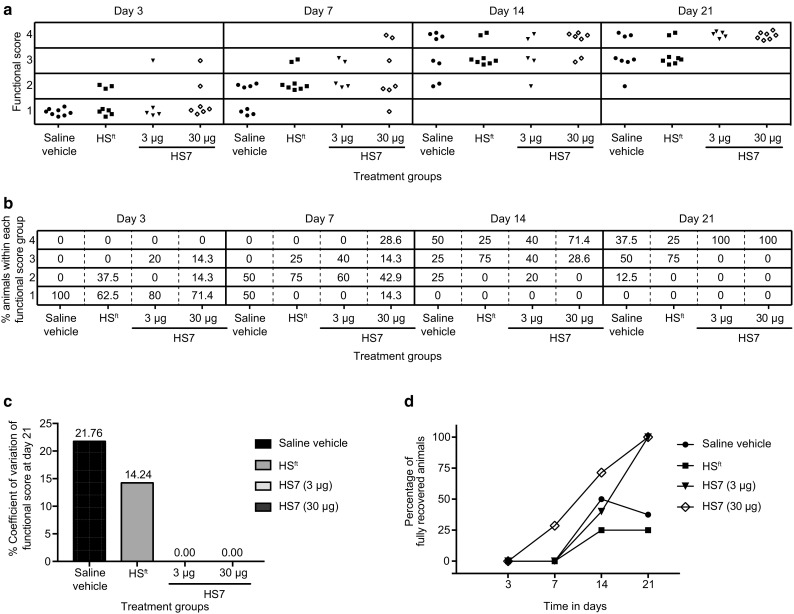
Ischaemic limb function scored on days 3, 7, 14 and 21 **a** Each symbol represents one animal in a scatter plot, which is represented numerically in **b** as percentage of animals. Animals were removed from the HS7 treatment groups if any abnormal abrasions hindered normal functional recovery. **c** Percentage coefficient of variation of the functional score on day 21. **d** Percentage of animals that showed full recovery (having a score of 4) on days 3, 7, 14 and 21

To determine any correlation between the blood volume re-established in the hindlimb and recovery of function, we utilised a functional recovery score index (Table 1) modified from a model described by Aitsebaomo et al. [24]. All animals were observed on Days 3, 7, 14 and 21 for functional use of the limb and plantar foot. Three animals in the 3 µg treatment group and one animal in the 30 µg treatment group were removed due to the presence of abrasions on their plantar foot that hampered normal functional recovery. The data showed that mice treated with HS7 recovered functional use of the ischaemic limb more rapidly compared to those treated with saline or HS^ft^ (Fig. 5a, b). On day 3, when the animals were still receiving their daily treatments, those in the HS7 groups showed increased restoration of function in their ligated limb. By day 7, which coincided with the last day of therapy, two mice receiving the highest amount of HS7 demonstrated full functional use of the ligated limb. In comparison, mice receiving saline or HS^ft^ had greatly reduced functional recovery in the affected limb. This observation was maintained throughout the study. The variability in functional recovery at day 21 across the various treatment groups was also assessed (% coefficient of variation) (Fig. 5c). The data show that treatment with HS7 coupled high precision with low variability (%CV = 0.0) in outcome compared to the increased variability resulting from treatment with vehicle (%CV = 21.76) or HS^ft^ (%CV = 14.24) (Fig. 5c). Also, the percentage of animals that achieved full functional recovery was consistently higher from day 7 in animals treated with HS7, compared to saline vehicle or HS^ft^ (Fig. 5d).

**Fig. 6:**
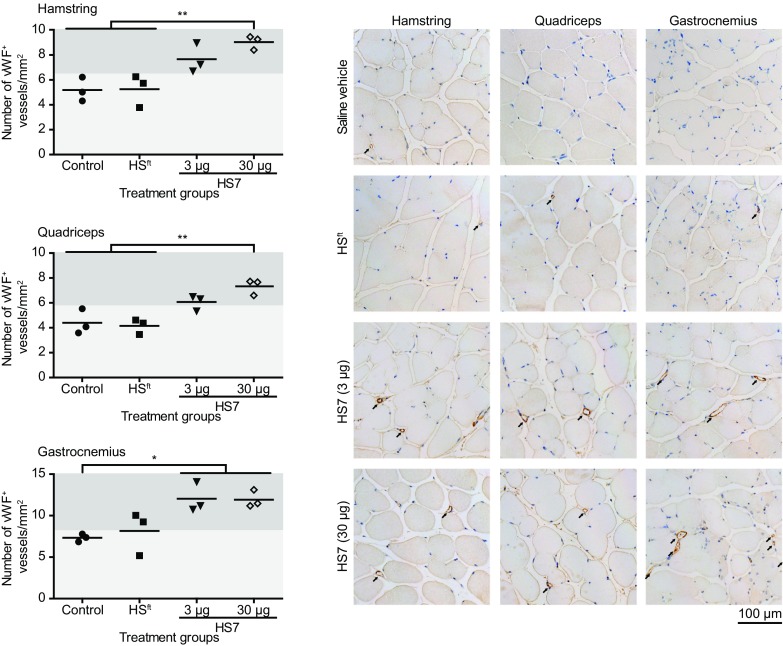
Histological assessments on hindlimb tissues harvested on day 8. The number of von Willebrand factor-stained vessels seen in cross section was counted from six random fields of view from images captured under brightfield using the Metafer 4 automated capture system and presented as mean number ± standard deviation per square millimetre (mm^2^). Representative cross-sectional images from hamstring, quadriceps and gastrocnemius are shown. For display purposes, representative images captured on ZEISS AxioImager Z1 were adjusted uniformly to increase the contrast of the reddish-brown stains on ImageJ. Arrows identify vessels positive for von Willebrand factor. Scale bar = 100 µm. (*p < 0.05; **p < 0.01)

The largest response in blood volume was observed on day 8, which coincided with the end of the treatment period. We performed an immunohistological analysis at this time point by staining for von Willebrand factor, a marker for endothelial cells that line the lumen of blood vessels, so enumerating the number of vWF^+^ vessels per mm^2^ in each muscle group (Fig. 6). The data were further stratified into two groups based on a threshold relative to the highest vessel density in the control group. The data showed that irrespective of the muscle group, ischaemic limbs treated with HS7 (30 µg) consistently had significant increases in the number of blood vessels compared to treatment with either saline or HS^ft^. Notably, treatment with HS^ft^ was comparable to the vehicle, except for two of the animals that had a slightly higher number of vWF^+^ vessels in the gastrocnemius muscle; however, this was still lower than treatment with HS7 (Fig. 6). In general, ischaemic sites treated with HS7 at 3 µg trended lower in vWF^+^ vessel number compared to those treated with 30 µg, except in the gastrocnemius muscle, where it was comparable.

Consistent with previous studies [25], our data showed that tissues in the lower extremity were most affected by PAD. We therefore further quantified the number of regenerating myofibres in the gastrocnemius muscle. We chose to examine responses to HS7 (30 µg) because the recovery of these animals was consistently higher than the other groups tested. The data showed that regenerating myofibres, characterised by centrally located nuclei [21], trended higher in their numbers in the HS7 group compared to saline controls (Fig. S2), further substantiating the positive effects observed with HS7 treatment. The number of αSMA^+^-vessels in the quadriceps was also 1.65-fold higher in the HS7 group (Fig. S3, *p < 0.05).

## Discussion

In this study, we demonstrate that treatment of ischaemic limbs with a blood vessel ECM-mimicking HS variant (HS7), one that avidly binds VEGF_165_, can accelerate subsequent revascularization and functional recovery. This particular HS7 variant was purified from HS^pm^ via affinity-chromatography targeting the heparin-binding domain of VEGF_165_.

VEGF_165_ is the most abundant isoform in its family and exists as a homodimer in vivo. When the homodimer binds two copies of its receptor, VEGF receptor-2, angiogenesis is initiated. Thus, maintaining the stability of the VEGF_165_ homodimer is central to unlocking the therapeutic effects of this potent angiogenic growth factor. Moreover, achieving this by targeting endogenously produced ligands represents a novel means of regulating the body’s angiogenic cascade. Data from the in vitro study demonstrate that HS7 binds and sustains homodimeric VEGF_165_ for up to 6 h, whereas complexation with a low-affinity binding HS variant (HS^ft^) did not improve the stability of the homodimer. That is, an HS variant with increased affinity for a particular growth factor can enhance the growth factor’s bioactivity. Similar increased stability in FGF-2 (fibroblast growth factor-2) [26] and VEGF [19] has been observed in the presence of heparin, a highly sulphated form of HS [27]; however, due to its powerful anticoagulant activity [28], the administration of heparin raises strong concerns about associated bleeding [29], let alone its myriad of ancillary, adverse clinical reactions. Heparin-bonded bypasses, or stent grafts for below-knee bypasses, have also brought about heparin-induced thrombocytopenia in patients [30]. Furthermore, while the high sulphation of heparin affords the ability to bind and stabilise a broad range of proteins, its propensity for indiscriminate binding also carries the substantial risk of activating various off-target biological processes, so leading to unfavourable healing outcomes.

In addition to thermal stability, studies in vitro show that HS7 also reduced VEGF_165_ susceptibility to proteolysis by plasmin, an enzyme present in injured tissue that plays several roles during angiogenesis, including the release of ECM proteins, activation of matrix metalloproteinases and modulating the availability of major angiogenic growth factors. VEGF-induced angiogenesis in plasminogen-deficient mice is known to be compromised, for example [31]. However, VEGF_165_ is also susceptible to plasmin, with a resultant reduction in its mitogenic activity by the removal of its heparin-binding domain [32]. Our results demonstrate that HS7 reduced VEGF_165_ susceptibility to plasmin, reemphasizing the importance of the heparin-binding domain of VEGF_165_ for its angiogenic activity, as others have previously indicated [23, 32, 33].

Our previous report showed that HS7 alone was not able to stimulate endothelial cell proliferation [18], thus making the selection of C57BL/6 mice for this proof-of-concept study crucial [9]. Also, the period of treatment coincided with the time-frame when VEGF is stably detected in the ligated tissue [9]. Based on our MRA data, even 3 µg of HS7 was sufficient to boost blood flow in the ischaemic limb and increase vWF^+^ vessel number by day 8. It was, however, insufficient to recover perfusion in the plantar foot as well as 30 µg of HS7. This appears to represent a dose–response criterion that has to be considered for future trials. The observation of more regenerating myofibres and αSMA^+^-vessels after only one week of treatment with HS7 was encouraging as it indicated that HS7 delivery could promote the recovery that was subsequently observed in our functional assessments of limb usage (Fig. 5). The presence of more αSMA^+^-vessels indicated the presence of mature vessels that could contribute to the higher blood flow quantified in HS7-treated animals. The comparison of HS^ft^ treatment outcomes was of particular interest. Based on our in vitro findings, HS^ft^ had much reduced stabilising effects on VEGF_165_. This might explain the minimal effect HS^ft^ had on accelerating blood reperfusion in the ischaemic limb, even though its interaction with other angiogenic factors upregulated during ischaemia seems highly likely. Recovery from ligation was similar to the saline group and could represent the normal rate of blood flow recovery that occurs in C57BL/6 mice [34, 35]. A concern with the use of HS7 as a treatment for vascular ischaemia was the potential for HS7 to bind to a host of other proteins in vivo via electrostatic interaction, or for proteins in serum to disrupt the HS7-VEGF_165_ interaction. Therefore, we deemed it encouraging when affinity-chromatography of FBS pre-loaded with both BMP-2 and VEGF_165_ demonstrated that HS7-tagged columns had a higher affinity for VEGF_165_ (Fig. 2b). This confirmed that the HS7-VEGF_165_ interaction was maintained despite the presence of proteins in serum that could potentially hinder this interaction. Such data lend support to our hypothesis that HS7 was able to bind and potentiate the endogenous ligands like VEGF_165_ to promote reperfusion recovery. However, while the HS7-column bound more VEGF_165_ by virtue of its increased affinity for the growth factor, it does not answer the question of whether they co-localise in vivo, which necessitates further investigations.

Recent reports have highlighted the possibility of HS-activating Toll-like receptor 4, so promoting inflammatory events that could initiate angiogenesis at ischaemic sites [36, 37]. Indeed, inflammation is observed in patients suffering from critical limb ischaemia with underlying atherosclerotic pathology [38]. However, our SPR data revealed no interaction between TLR4 and heparin (Fig. S4a) immobilised on a SA chip support, in contrast with the experiments that showed VEGF_165_ binding to immobilised heparin in a dose-dependent manner. Incubation of RAW264.7 with HS also showed that the TLR4 signal transduction pathway was not activated, whereas phosphorylation was observed when cells were exposed to LPS as a positive control (Fig. S4b).

The use of HS on its own for the treatment of limb ischaemia is in contrast to studies investigating gene-, protein- or cell-based treatments for therapeutic angiogenesis in similar animal models [34, 39–41]. The assumptions of such studies still rely on increasing the local concentration of angiogenic growth factors to stimulate neovascularization. In contrast, similar studies in cutaneous wounds [42] and pressure ulcers [43] in rats using the HS glycosaminoglycan mimetic OTR4120 support the finding that external applications of HS potentiate the endogenous healing capacity of tissues experiencing damage. The administration of another synthetic glycosaminoglycan, OTR4131, accelerated neovascularization in rat limb ischaemia as well as muscle regeneration [44]. These and other studies postulated that the binding of synthetic glycosaminoglycans to VEGF might potentiate the growth factor’s angiogenic activity [45] through a combination of mechanisms shown by our in vitro assays in the current report, as well as from past investigations [18]. We show here that HS7 prolongs the active, dimeric VEGF_165_ for a longer period than the low-affinity HS^ft^ or in its absence. Also, the use of an affinity-tuned HS to enhance the activity of VEGF_165_ should decrease the risk of off-target effects of HS, as seen in the case of heparin, which binds non-discriminately to a large number of proteins. Synthetic glycosaminoglycans may replace endogenous HS species that are degraded in wound sites, but they presumably bind a large number of factors, including agonists and antagonists, unless they are carefully filtered. Naturally occurring HS species carry targeted binding motifs that synthetic GAGs cannot fully mimic.

Using a murine hindlimb ischaemia model, we showed that HS7 improved blood flow recovery. Despite the positive outcomes, we recognise the inherent limitations of the study. One of them is the use of 10-week old C57BL/6N mice, which is akin to replicating the presence of active repair cascades that occur in young, healthy adolescents or adults [46]. PAD and critical limb ischaemia are more prevalent in aged and unhealthy populations, such as patients with diabetes [47, 48]. It is therefore crucial that the efficacy of HS7 is further validated in animals models with accompanying comorbidities [46]. For HS7 to be a suitable therapeutic agent, amounts of endogenous VEGF expression in the patient become crucial. Healing capacity is observed to decrease with age in a mouse wound model, due to reduced gene expression of growth factors and their receptors [49]. The combined delivery of VEGF_165_ and HS7 may still be required in some patients. HS7 stabilisation of ligands also offers the additional advantage of reducing the high doses of exogenous growth factor required, providing a possible solution to the side effects that accompany the use of growth factors in revascularization therapy.

## Electronic supplementary material

Below is the link to the electronic supplementary material.

Fig. S1—Elution of VEGF_165_ from HS-tagged columns The affinity-chromatography of growth factors from FBS using HS-tagged columns was performed twice. Densitometry analysis of each replicate was performed separately and represented here as “Replicate 1” (shown in Fig. 2B) and “Replicate 2”. In both replicates, HS7-tagged columns captured higher amounts of VEGF_165_ compared to the HS^ft^-tagged columns (EPS 1498 KB)

Fig. S2—Comparison of number of regenerating myofibres The total number of regenerating myofibres from H&E stained sections in a cross section of gastrocnemius tissue were counted and presented as mean number ± standard deviation per square millimetre (mm^2^). Values were from two independent observers. The bottom panel shows representative cross section images from saline vehicle and HS7 (30 µg) groups. Regenerating myofibres were identified by their centrally located nuclei. White arrows indicate some of the regenerating myofibres. Scale bar = 100 µm. Treatment group size: n = 3 (EPS 74233 KB)

Fig. S3—Comparison of number of αSMA^+^ vessels The number of αSMA^+^ vessels from quadriceps was counted from random field-of-views and presented as mean number ± standard deviation per square millimetre (mm^2^). The observer was blinded to the treatment the animals received. Treatment group size: n = 3 (EPS 433 KB)

Fig. S4—Effect of heparin and HS on TLR4 (a) SPR curves showed no interaction of TLR4 with heparin immobilised on the sensor chip; in comparison, VEGF_165_ showed dose-dependent interaction on the same SPR chip. (b) HS (10 µg/ml) was incubated with the murine macrophage cell line RAW264.7 and phosphorylated proteins in the TLR4-signalling cascade were probed. Two replicates of the experiment were performed and represented here as “Replicate 1” and “Replicate 2”. Both replicates demonstrated similar findings (EPS 6442 KB)

Table S1 (DOCX 104 KB)
