# Supplementary material for: Improved recovery from limb ischaemia by delivery of an affinity-isolated heparan sulphate
Source: Angiogenesis. 2018 May 18;21(4):777–91. doi: 10.1007/s10456-018-9622-9 (PMC6208897; doi:10.1007/s10456-018-9622-9)
Supplement: Supplementary file 5 — Table S1 (DOCX 104 KB) [file 10456_2018_9622_MOESM5_ESM.docx]

**Table S1: List of animals**

| Animal ID | Treatment | LDI * | MRA * | Histology * |
| --- | --- | --- | --- | --- |
| S2 | vehicle | -2, 3, 7, 14, 21 | 1, 8, 15, 22 | ✗ |
| S4 | vehicle | -2, 3, 7, 14, 21 | 1, 8, 15, 22 | ✗ |
| S5 | vehicle | -2, 3, 7, 14, 21 | 1, 8, 15, 22 | ✗ |
| S6 | vehicle | -2, 3, 7, 14, 21 | 1, 8, 15, 22 | ✗ |
| S7 | vehicle | -2, 3, 7, 14, 21 | -3, 1, 8, 15, 22 | ✗ |
| S8 | vehicle | -2, 3, 7, 14, 21 | 1, 8, 15, 22 | ✗ |
| S18 | HS7 (30 µg) | -2, 3, 7, 14, 21 | 1, 8, 15, 22 | ✗ |
| S19 | vehicle | -2, 3, 7, 14, 21 | 1, 8, 15, 22 | ✗ |
| S20 | HS7 (30 µg) | -2, 3, 7, 14, 21 | 1, 8, 15, 22 | ✗ |
| S21 | HS7 (30 µg) | -2, 3, 7, 14, 21 | 1, 8, 15, 22 | ✗ |
| S22 | HS7 (3 µg) | -2, 3, 7, 14, 21 | 1, 8, 15, 22 | ✗ |
| S23 | HS7 (3 µg) | -2, 3, 7, 14, 21 | 1, 8, 15, 22 | ✗ |
| S24 | HS7 (3 µg) | -2, 3, 7, 14, 21 | 1, 8, 15, 22 | ✗ |
| S25 | HS7 (30 µg) | -2, 3, 7, 14, 21 | 1, 8, 15, 22 | ✗ |
| S26 | HS7 (3 µg) | -2, 3, 7, 14, 21 | 1, 8, 15, 22 | ✗ |
| S27 | HS7 (30 µg) | -2, 3, 7, 14, 21 | -3, 1, 8, 15, 22 | ✗ |
| S28 | HS7 (3 µg) | -2, 3, 7, 14, 21 | -3, 1, 8, 15, 22 | ✗ |
| S30 | HS7 (30 µg) | -2, 3, 7, 14, 21 | 1, 8, 15, 22 | ✗ |
| S31 | HS7 (3 µg) | -2, 3, 7, 14, 21 | -3, 1, 8, 15, 22 | ✗ |
| S34 | HS7 (3 µg) | -2, 3, 7, 14, 21 | -3, 1, 8, 15, 22 | ✗ |
| S35 | HS7 (30 µg) | -2, 3, 7, 14, 21 | 1, 8, 15, 22 | ✗ |
| S36 | HS7 (3 µg) | -2, 3, 7, 14, 21 | 1, 8, 15, 22 | ✗ |
| S37 | HS^ft^ | -2, 3, 7, 14, 21 | 1, 8, 15, 22 | ✗ |
| S38 | HS^ft^ | -2, 3, 7, 14, 21 | 1, 8, 15, 22 | ✗ |
| S39 | HS^ft^ | -2, 3, 7, 14, 21 | 1, 8, 15, 22 | ✗ |
| S40 | HS^ft^ | -2, 3, 7, 14, 21 | -3, 1, 8, 15, 22 | ✗ |
| S41 | HS7 (30 µg) | -2, 3, 7, 14, 21 | 1, 8, 15, 22 | ✗ |
| S42 | HS^ft^ | -2, 3, 7, 14, 21 | 1, 8, 15, 22 | ✗ |
| S43 | HS^ft^ | -2, 3, 7, 14, 21 | -3, 1, 8, 15, 22 | ✗ |
| S44 | vehicle | -2, 3, 7, 14, 21 | -3, 1, 8, 15, 22 | ✗ |
| S45 | HS^ft^ | -2, 3, 7, 14, 21 | 1, 8, 15, 22 | ✗ |
| S46 | HS^ft^ | -2, 3, 7, 14, 21 | 1, 8, 15, 22 | ✗ |
| A3 | vehicle | ✗ | ✗ | 8 |
| A5 | HS7 (3 µg) | ✗ | ✗ | 8 |
| B1 | HS7 (30 µg) | ✗ | ✗ | 8 |
| B3 | HS^ft^ | ✗ | ✗ | 8 |
| B4 | vehicle | ✗ | ✗ | 8 |
| C1 | HS7 (3 µg) | ✗ | ✗ | 8 |
| C2 | HS7 (30 µg) | ✗ | ✗ | 8 |
| C3 | HS^ft^ | ✗ | ✗ | 8 |
| C4 | vehicle | ✗ | ✗ | 8 |
| D2 | HS7 (3 µg) | ✗ | ✗ | 8 |
| D3 | HS7 (30 µg) | ✗ | ✗ | 8 |
| D4 | HS^ft^ | ✗ | ✗ | 8 |

* Numbers indicate the day when data for LDI or MRA was collected, or when animals were sacrificed for histology sample collection. A cross (✗) indicates that data or sample was not collected.
